# Gene Expression Suggests Spontaneously Hypertensive Rats May Have Altered Metabolism and Reduced Hypoxic Tolerance

**DOI:** 10.2174/156720212799297074

**Published:** 2012-01

**Authors:** Marie-Françoise Ritz, Caspar Grond-Ginsbach, Stefan Engelter, Philippe Lyrer

**Affiliations:** 1 Department of Biomedicine, Brain Tumor Biology Laboratory, Pharmazentrum, Klingelbergstrasse 50, 4056 Basel, Switzerland; 2 Neurology Clinic, University of Heidelberg, Im Neuenheimer Feld 400, 69120 Heidelberg, Germany; 3 University Hospital, Stroke Unit, Neurology Clinic, Petersgraben 4, 4031 Basel, Switzerland

**Keywords:** cDNA microarrays, energy metabolism, hypoxia, small vessel disease, SHR, WKY.

## Abstract

Cerebral small vessel disease (SVD) is an important cause of stroke, cognitive decline and vascular dementia (VaD). It is associated with diffuse white matter abnormalities and small deep cerebral ischemic infarcts. The molecular mechanisms involved in the development and progression of SVD are unclear. As hypertension is a major risk factor for developing SVD, Spontaneously Hypertensive Rats (SHR) are considered an appropriate experimental model for SVD. Prior work suggested an imbalance between the number of blood microvessels and astrocytes at the level of the neurovascular unit in 2-month-old SHR, leading to neuronal hypoxia in the brain of 9-month-old animals. To identify genes and pathways involved in the development of SVD, we compared the gene expression profile in the cortex of 2 and 9-month-old of SHR with age-matched normotensive Wistar Kyoto (WKY) rats using microarray-based technology. The results revealed significant differences in expression of genes involved in energy and lipid metabolisms, mitochondrial functions, oxidative stress and ischemic responses between both groups. These results strongly suggest that SHR suffer from chronic hypoxia, and therefore are unable to tolerate ischemia-like conditions, and are more vulnerable to high-energy needs than WKY. This molecular analysis gives new insights about pathways accounting for the development of SVD.

## INTRODUCTION

Cerebral small vessel disease (SVD) is characterized by lacunar infarcts and diffuse, ischemic white matter lesions (leukoaraiosis) affecting subcortical, periventricular or pontine regions, dilated perivascular spaces, and microbleeds. SVD is associated with focal motor deficits, and cognitive decline that often progress to dementia. Hypertension has been shown to be an important risk factor for SVD and in the development of VaD [1]. The analysis of animal models of hypertension in which brain damage is documented can provide useful information about genes and pathways underlying pathophysiological traits in humans. Hypertension in SHR impairs cerebrovascular autoregulation and CBF responses and aggravates tissue damage after focal brain ischemia [2,3]. SHR experience brain atrophy, loss of nerve cells in cerebrocortical areas, and glial reaction. Small vessels in the cerebral cortex play crucial roles in the maintenance of normal tissue blood flow and are the most susceptible to vascular dysfunction caused by hypertension. The small intraparenchymatous cerebral arteries, including resistance arteries, are constitutively exposed to hemodynamic forces in this rat strain. Consequently, SHR are more susceptible to endothelial dysfunction caused by hypertension and are prone to subsequent SVD such as lacunar infarctions, spontaneous intracerebral hemorrhage, and cognitive impairment with leukoaraiosis. Cognitive data for these animals are spare, however, impaired learning and memory were observed coincident with increased anxiety [4,5].

Anatomical differences in the brains of SHR were already described previously, such as hypertrophy of the arterial wall of large arteries [6]. In a previous study, we detected cerebral abnormalities at the neurovascular unit (NVU) such as enhancement of the number of the capillaries and venules and reduction of the number, or atrophy of astrocytes in SHR when compared to age-matched normotensive Wistar Kyoto (WKY) controls [7]. The differences observed in the densities of capillary and of the astrocytic contingent were already significant in young pre-hypertensive, 2-month-old SHR and in stroke-prone SHR (SP-SHR), as well as in adult 9-month-old animals. This raises the question whether factors other than hypertension, such as genetic predispositions, induce cellular dysfunction that may contribute to the increased susceptibility to brain hypoxia in these rat strains.

In the NVU, astrocytes are the major intermediary cells linking vessels and neurons [8]. They provide neuronal nutrition, maintain ionic, neurotransmitter, and metabolic homeostasis, and contribute to neuroimmune functions. It is not known if a disturbance of the vessel-astrocyte interaction causes hypoxia, although it is well described that general hypoxia induces the accumulation of hypoxia-inducible factor HIF-1α in rat brain tissue [9]. Accumulation of HIF-1α was observed in neurons of 9-month-old SHR and SP-SHR, but not in rats aged 2 months, suggesting that the cellular imbalance between astrocytes and blood vessels reduces exchanges of nutrients and oxygen between blood and brain tissue, causing the development of hypoxia-like conditions with time. 

Given the crucial role of astrocytes in maintaining ionic, neurotransmitter, and metabolic homeostasis in the brain, we need a better molecular understanding of the mechanisms inducing this imbalance between microvessels and astrocytes observed in the brains of young SHR.

Oligonucleotide-based microarray technology is a powerful way to gain insight into the molecular pathogenesis of the disease. Gene expression profiling may allow identification of new players in pathological processes such as small vessel disease. This method has been used to detect genes differentially expressed between SHR and WKY, involved in the development and maintenance of hypertension in SHR. Since renal function have been recognized as a prerequisite for the maintenance of all forms of hypertension [10], these gene expression analysis have been performed using the kidneys as starting material.

Using this technology, we examined the differences in gene expression profiles in the cortex of young pre-hypertensive and hypertensive SHR rats versus control normotensive WKY. The genes identified with this approach may provide guide to potential therapeutic targets for neuroprotection to be used in the prevention of small vessel disease in patients at risk.

## MATERIAL AND METHODS

### Animals and Brain Tissue Collection

The procedures used for the care and euthanasia of the animals followed the "Principles of laboratory animal care" (NIH publication No. 86-23, revised 1985) and were approved by the Animal Welfare Committee Canton Basel Land and in compliance with the Swiss Guidelines for the Care and Use of Animals. Four (4) spontaneously hypertensive rats (SHR, 2 aged 2 months, 2 aged 9 months) and 4 Wistar Kyoto rats (WKY, 2 aged 2 months, 2 aged 9 months) were purchased from Charles River Laboratories (Sulzfeld, Germany) and housed in regulated environment with free access to food and water. SHR are obtained from outbred WKY male with marked elevation of blood pressure mated to female with slightly elevated blood pressure. After euthanasia (by decapitation following anesthesia with isoflurane), the brains were rapidly removed and immediately frozen at -80°C. Brains were mounted in a cryostat and coronal sections were made, until the frontal cortex was reached (approx. 4 mm anterior to the bregma). Samples were then taken using a tissue corer (2 mm diameter, 1 mm length) and kept frozen until RNA extraction. 

### RNA Preparation and Expression Profiling

The frozen brain tissue samples were homogenized in Purezol solution (Bio-Rad). Isolation of total RNA from the lysate including DNAse I treatment were carried out using the BioRad Aurum Total RNA Fatty and Fibrous Tissue kit (BioRad) according to the manufacturer’s protocol. The purity and quality of the extracted total RNA were determined using the Agilent 2100 Bioanalyzer (Agilent Technologies, Santa Clara, CA, USA) and the NanoChip protocol before microarray experiments. Double strand cDNA was synthesized from 1 µg of total RNA using a cDNA Synthesis System (InvitroGen, Basel, Switzerland) with the T7-(T)_24_ primer. The *in vitro*-labeling kit (Enzo; Farmingdale, NY) was used to transcribe the cDNA into cRNA in the presence of Biotin-11-CTP and Biotin-16-UTP, according to the kit instructions. After purification with the MinElute kit (Qiagen; Hilden, Germany), integrity of the cRNA were assessed by the BioAnalyzer (Agilent Technologies, Palo Alto, CA). Twelve (12) µg fragmented cRNA was then used for hybridization to the Affymetrix GeneChip^®^ Rat Gene 1.0 ST Arrays (Affymetrix, Santa Clara, CA, USA). Sample labeling, hybridization, scanning and data outputs were performed according to the manufacturer’s protocols in collaboration with the Life Sciences Training Facility, Division of Molecular Psychology, University of Basel, Switzerland. 

## DATA ANALYSIS

Microarray data generated from SHR were directly compared to those from WKY, for each age group separately. Data expression values were collected as individual CEL files for each donor from the Rat Gene 1.0 ST Arrays and were then normalized in XRAY (XRay^®^ statistical program, Biotique Systems, Inc., www.biotiquesystems.com) with full quantile normalization. The arrays were normalized as a single file in XRAY comprised of 8 individual CEL files. Data analysis including background subtraction, normalization, and elimination of false positives was performed using the default parameters. Mixed model analysis of variance (ANOVA) was used to identify genes differentially expressed that exhibited significant changes in signal levels with a *P-*value less than 0.05 and fold changes greater than 2. 

The principal component analysis (PCA) of all samples was performed using Partek^®^ Genomics Suite^TM^ (Partek GS, Partek Inc., St. Louis, MO, USA) to assess variability of the data across rat strains and ages. 

### Microarray Validation by Quantitative Real-Time PCR (qRT-PCR)

The microarray results were confirmed by using quantitative real-time polymerase chain reaction (qRT-PCR) for 3 selected genes that demonstrated significantly altered gene expression common between the 2- and 9-month-old animals: retinol saturase (*Retsat*), CD151 antigen, and succinate dehydrogenase complex (*Sdha*). Glyceraldehyde 3-phosphate dehydrogenase (*Gapdh*) was used as housekeeping gene. The primers were designed from unique site over exon-intron junctions to prevent amplification of genomic DNA, and the primers for *Gapdh* were purchased from Qiagen (Hilden, Germany, cat. N° QT00199633). Primer sequences were searched against BLAST to ensure that they did not match any known gene aside from that for which they were designed (especially other family members) and are as follows (5’ to 3’): CD151 (Genebank NM_022523): CAGTCTGCCTCAAGTATCTG and TGTAGGCTGTTGC TAGGTAG; Retinol saturase (Genebank NM_001034126): GTGGGAGGAAAGAATACATC and TCAGTAGGATGG CATGAGAG; succinate dehydrogenase (Genebank NM_ 198788): ACAAAGCTCTTTCCTACCCG and TAATGGA TGGCATCCTGATC. First strand cDNA was created from 1 µg of total RNA using the iScript cDNA synthesis kit from Bio-Rad. Quantitative RT-PCR was performed on 1 ng of total cDNA per well. Each sample was run in triplicate on the Bio-Rad detection system (C1000 and CFX96, Bio-Rad, Reinach, Switzerland). Each measurement was made in triplicate and expressed relative to the internal control GAPDH. The comparative cycle threshold (C(t)) method was used to calculate relative quantification of gene expression. The following formula was used to calculate the relative amount of the transcripts in the SHR samples and the WKY samples, and both were normalized to the endogenous control (GAPDH): ΔΔC(t)=ΔC(t)SHR-ΔC(t)WKY. ΔC(t) is the difference in C(t) between the target gene and endo-genous control. The fold-change for the SHR samples relative to WKY samples was calculated by 2E-ΔΔC(t). Statistically significant changes were identified by a two-tailed *t*-test with a *P*-value less than 0.05.

## RESULTS

### Gene Expression Analysis

**Fig. (1):**
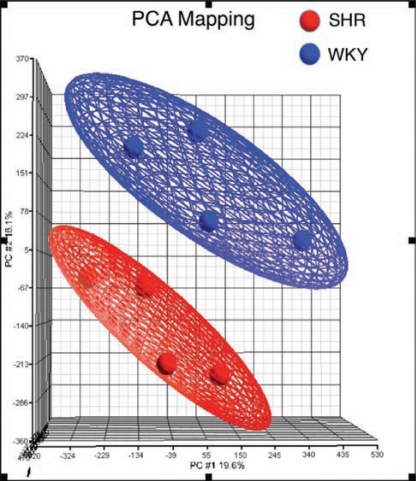
3D representation of the principal components analysis (PCA) of gene expression profiles in the frontal cortex of SHR and WKY. The intensity of the entire gene set was used. Each point in the graphic represents a microarray chip and points that are near each other denote characteristics that are similar across the entire genome.

In this study, brain frontal cortical areas from 2 and 9-month-old SHR and WKY were selected for RNA isolation and microarray analysis. Principal Components Analysis (PCA) of global gene expression in the 8 rats (4 SHR and 4 WKY) demonstrated two apparent clusters with the samples separating well into SHR and WKY groups (Fig. **1**). In each group, an additional age-dependent segregation was visible (not shown on the figure). 

Data filtering identified subsets of genes with significant changes in the expression in these 2 rat strains, as summarized in Table **1**. In the 2-month-old group a total of 156 genes were differentially expressed between both rat strains. Among these genes, 58 were upregulated and 98 were down-regulated in SHR compared with WKY. In the 9-month-old group, a total of 131 genes were differentially expressed, with 69 genes up-regulated and 62 genes down-regulated in SHR. 

### Common Genes Differentially Expressed in both Groups of Ages between SHR and WKY

Comparing the lists of genes differentially expressed in 2 and 9-month-old animals, we found 18 common genes. Among them, 10 genes were upregulated, and 7 were downregulated in SHR compared with WKY. One gene was upregulated in the 2-month-old group and downregulated in the 9-month-old group (ubiquitine-protein ligase E3). 

These genes differentially expressed could be clustered in different cell functions or biological processes as listed in Table **2**: vascular development and endothelial proliferation, CD151, Activin A receptor, p21-activated kinase 2 (*Pak2*), genes that were significantly overexpressed in SHR; mitochondrial function and structure: cytochrome c oxidase subunit VIIa polypeptide 2 like (*Cox7a2l*), found overexpressed in SHR, and ribosomal protein L17 (*Rpl17*), that was found underexpressed; energy and lipid metabolisms: succinate dehydrogenase complex a (*Sdha*) downregulated, and acyl-coenzyme A dehydrogenase (*Acadsb*) upregulated; oxidative stress responses: retinol saturase (*Retsat*), nitrilase family (*Nit*), glutathione S-transferase (*Gs*) both downregulated, Heat shock protein 40 (*Hsp40*, also called *DnaJ*), upregulated; and protein catabolism: ubiquitin-activating enzyme E1 (*Ube1*), upregulated, and ubiquitin-protein ligase E3A (*Ube3a*) upregulated in 2-month-old SHR but down-regulated in 9-month-old SHR. Five other genes with miscellaneous functions were also found differentially expressed between SHR and WKY.

### Quantitative PCR Validation of the Microarray Results

To confirm results obtained by Affymetrix arrays, selected genes from different biological functions in the list of differentially expressed genes in common in the 2 and 9-month-old SHR (Table **2**) were analyzed by quantitative RT-PCR against cDNA derived from four individual mice per group. Particular focus on genes involved in each of the functional process such as vessel proliferation (CD151 antigen), energy metabolism (succinate dehydrogenase complex a, *Sdha*), and oxidative stress (retinol saturase, *Retsat*) were chosen using specific primers. As shown in Fig. (**2**), quantitative RT-PCR confirmed the direction of changes seen on the gene microarrays for these genes when comparing age-matched SHR with WKY.

### Genes Differentially Expressed Specifically in the 2-Month-Old Group

In addition to the common genes differentially expressed in SHR compared with WKY in both ages, we found many genes significantly differentially expressed only in 2-month-old SHR compared with age-matched WKY (Table **3**). Notably, a cluster of genes for mitochondrial metabolic enzymes emerged, comprising beta-oxidation of fatty acids, the tricarboxylic acid cycle, and respiratory chain complexes phosphorylation, as well as mitochondrial ribosomal proteins. Although the expression of methylmalonyl coenzyme A mutase was repressed, the expression of 2 genes belonging to oxidative respiration (cytochrome c oxidase, *Cox*, and NADH dehydrogenase Fe-S protein 7, *Ndufs7*) were higher in SHR. Interestingly, the gene for the enzyme glycogen phosphorylase, *Pygb*, was also found overexpressed in the 2-month-old SHR. There was also an increased expression of 4 genes involved in anti-oxidant defenses and in the response to stress in SHR compared with WKY. In these young animals, the expression of a set of 4 genes participating in the development and function of the CNS was significantly downregulated in SHR.

### Genes Differentially Expressed in the 9-Month-Old Group Only

In this group, we further found abnormal expression of some genes involved in lipid metabolism (carnitine palmitoyltransferase 1c, *cpt1c*, acetyl-CoA carboxylase a, *Acaca*, and glycerol -3 phosphate acyltransferase, *Gpam*) and oxidative phosphorylation (Table **4**). In addition, an important group of genes inducing protein ubiquitination (ubiquitin-activating enzyme E1-domain containing 1, *Ube1dc1, *

*Praja2,* *Sumo2*) and degradation (dipeptidylpeptidase 10, *Dpp10*) was found mostly overexpressed in older SHR, as well as a number of genes involved in cell death (death effector domain-containing, *Dedd*, beclin1,* Becn1*, macro-phage migration inhibitor factor, *Mif*, and colony stimulating factor 1 receptor, *Csf1r*) not found in younger animal. Reduced expressions of genes encoding proteins responsible for ion homeostasis (ion channels, ATPases), neurotransmitter receptors (glutamate receptor, *Grinl1a*), synaptic signaling (neural stem cell-derived dendrite regulator, *Dsddr*), and other CNS functions were observed in older SHR.

### Genes Differentially Expressed Amongst 2- and 9-Month-Old SHR

This analysis comparing gene expression profiles between 2 and 9-month-old SHR was aimed to give some indication about the genes differentially expressed during aging of hypertensive animals. We found 124 genes significantly differentially expressed between 2- and 9-month-old SHR, 48 being more expressed in 2-month-old SHR and 76 being more expressed in 9-month-old SHR. A selection of differentially genes is given in Table **5**. Surprisingly, we found only one gene, coding for adducin 2-beta, involved in the development of hypertension in the brain. This gene was overexpressed in 2-month-old SHR. Other genes significantly differentially expressed were coding for ion channels, involved in nervous system development, all of them showing higher expression in 2-month-old SHR. Concerning apoptosis, a gene whose expression is thought to inhibit apoptosis (PDZ domain containing RING finger 3*, Pdzrn3*, also known as E3 ubiquitin-protein ligase) was more expressed in 2-month-old SHR. In contrast, several genes coding for factors inducing apoptosis (SCF apoptosis response protein 1, Programmed cell death protein 11, and Septin 4) were more expressed in 9-month-old SHR. 

## DISCUSSION

In the present study, the comparison of gene expression in SHR with WKY was performed in order to identify genes susceptible to elicit the pathogenesis of SVD. We are aware that dividing the groups of rats depending on their age limited the sample size (n=2) in each age-driven groups. However, the appearance of some identical genes differen- tially expressed in both groups of age in the SHR *vs*. WKY comparisons may strengthen the fact that these genes were not emerging by chance. In addition, the comparison of young with older SHR that identified genes involved in apoptotic pathways indicated that the number of samples is still reflecting relevant age effects. Some genes specifically expressed in the young (2 months) or the old (9 months) animals also appeared. In addition, this grouping also gave us some hints about the early deregulations of gene expression in young pre-hypertensive rats, in which the differences may not result from hypertension, but from genetic and/or anatomical changes compared to age-matched normotensive control animals. 

The potential relevance of some of the observed molecular differences highlighted in the present study is discussed below.

### Vascular Remodeling, Nervous System Development, Synaptogenesis

Consistent with our previous immunohistological analysis of the brain of SHR showing capillary proliferation in the brain of SHR [7], microarray data indicated that signals for angiogenesis are stimulated in the cortex of pre-hypertensive as well as hypertensive SHR. At least 2 genes involved in endothelial proliferation were significantly overexpressed, coding for CD151 antigen and activin A receptor. Brain hypoxia is known to induce angiogenic factors such as angiopoietin 2 and vascular endothelial growth factor (VEGF), thereby complementing the possible adaptation of energy metabolism and brain cell membrane ionic homeostasis. An angiogenic adaptive process seems to be switched on in SHR, and might be indicative of a chronic status of brain hypoperfusion [11]. 

Genes involved in the development of the nervous system showed increased expression levels in 2-month-old SHR compared to older SHR: Pleiotrophin (PTN), is a neurite growth promoting factor for the CNS development that can be induced in the adult CNS by ischemic insults [12]. Interestingly, this gene is upregulated in macrophages, astrocytes and endothelial cells in areas of ischemia-induced developing neovasculature. Because PTN is a potent angiogenic agent *in vitro* and *in vivo* [13], the increase in *Ptn* gene expression may be involved in the higher density of small blood vessels observed in the brains of young SHR. The expression of neuronal growth regulator 1 (Negr1), a cell adhesion protein involved in the trans-neural growth-promoting factor in regenerative axon sprouting in the mammalian brain, was recently found to be upregulated by stroke [14]. These relations between increased expression during stroke or ischemic condition strongly suggest that overexpression of these genes is an indicator of hypoxia in the brains of young SHR.

Axonal sprouting and changes in the dendritic spines have been characterized as potential mechanisms for plasticity and self-repair after an ischemic injury to the adult brain. Comparing 2-month-old SHR with 9-month-old SHR, *Copine IV* was the gene showing the strongest difference with lower expression in 9-month-old animals. This protein is specifically expressed in the brain and is involved in dendritic development. Post-stroke axonal sprouting remapping the connections of the somatosensory cortex has been observed [15], but ischemic preconditioning also promotes these processes [16]. Since copines affect spine morphology, changes in their expression could contribute or be involved in neurodevelopmental disorders. Indeed, deformed dendritic spines and dendritic retraction are hallmarks of many neurological conditions, notably in diseases in which cognitive performance is impaired [17]. Dendritic spine loss is reported in non-Alzheimer’s type dementias, and may represent a pathological acceleration of the normal decrease observed in senescence. In addition, two genes involved in axonal injury were differentially expressed in 9-month-old SHR: *Spectrin* *b3* was underexpressed, while *Calpain 7* was overexpressed. The calpain protein cleaves spectrin, therefore destroying cytoskeleton and leading to calpain-mediated axonal demise as shown after traumatic brain injury [18]. The reduced expression of Copine IV, and Spectrin b3 together with an elevated expression of Calpain 7 in 9-month-old SHR may participate in the cortical tissue atrophy observed in hypertensive animals.

### Mitochondrial Complexes and Energy Production 

In order to meet a sudden increase in the local energy demand, the brain tissue utilizes its stored energy in the form of glycogen breakdown as observed by a decrease in the glycogen levels in both liver and brain which is accompanied by a marked increase in the activity of glycogen phosphorylase, PYG. The activity of this cytoplasmic enzyme is present mostly in astrocytes [19] and is positively regulated by AMP and negatively regulated by ATP, ADP, and glucose-6-phosphate. Its overexpression in 2-month-old SHR compared to age-matched WKY therefore suggests that brain cells in these animals have to mobilize their fuel reserves to enhance fluxes in glycolytic and oxidative pathways. Overexpression of *Pyg* may therefore denote an adaptative mechanism leading to protect energetically compromised neurons in young SHR.

Mitochondria have a central function in the energy metabolism, and play important roles in generation of free radicals, ATP formation, and in apoptosis. Mitochondrial dysfunction, understood as the disruption of one or more of the events necessary to produce energy, has the immediate adverse consequence of energy failure, but also produces a massive increase in oxidative stress. Decreased ATP production and increased ROS production have been reported in various organs such as heart, liver and spleen of SHR [20]. In the brain of SHR, previous studies have shown clear signs of mitochondrial dysfunctions such as increased Ca^2+^ accumulation capacity [21], decreased energy production [22], and assembly defects in respiratory complexes at the protein level [23], that have been proposed to contribute to the pathogenesis of hypertension [24]. Proteomic approaches have shown that perturbation of a single complex can be enough to disrupt the integrity of neighboring complexes within the respiratory chain [23]. The present study high-lights some of the genes that may be involved in these defects, such as NADH dehydrogenase Fe-S protein 7 (complex I) and cytochrome c oxidase subunit VII polypeptide 2 (complex II). These two genes were overexpressed in 2- and 9-month-old SHR, leading probably to disequilibrium in the mitochondrial complexes, or a compensatory phenomenon aiming to improve energy production. Indeed, several genes coding for enzymes of the tricarboxylic acid (TCA) cycle occurring in the matrix of mitochondria and responsible for the generation of usable energy are also differentially expressed in young and old SHR: succinate dehydrogenase (*Sdha*) was found underexpressed in 2- and 9-month-old SHR. This mitochondrial enzyme complex participates to both the TCA cycle and the respiratory chain: it catalyzes the oxidation of succinate to fumarate in the TCA cycle and carries electrons to the ubiquinone pool of the respiratory chain. Deficiency of SDHA has been shown to be associated with a specific leukoencephalopathic syndrome [25,26]. Moreover, the heme group of this enzyme prevents the production of reactive oxygen species, therefore reducing oxidative stress. A reduced expression in SHR may therefore have severe consequences in terms of energy production and cellular stress.

### Lipid Metabolism

As in human with monogenic disorders of blood pressure regulation, SHR show hypertension-associated metabolic lipid and glucose disturbances [27]. However, changes in expression of genes involved in the incorporation of fatty acids have also been observed in young, pre-hypertensive SHR, and particularly a decreased expression of a gene responsible for the synthesis of long-chain fatty acyl-CoAs (Acyl-CoA long-chain family member 3, *Acsl3*), suggesting that these differences in gene expression are hypertension-independent. Young and older SHR also showed increased expression of the gene coding for the fat metabolizing enzyme acyl-CoA dehydrogenase family (*Acad9*) acting during the first step of mitochondrial beta oxidation of fatty acids vital for energy production in periods of fasting and other metabolic stress. Young SHR showed decreases in the expression of long-chain acyl CoA member 3 (*Acsl3*) and carnitine O-octanoyltransferase genes. An underexpression of methylmalonyl Coenzyme A mutase (*Mcm*) has also been observed in 2-month-old SHR. This enzyme generates succinyl-CoA, a key molecule of the TCA cycle. Older SHR showed decreased expression in glycerol 3-phosphate acyl-transferase (*Gpa*), and acetyl CoA carboxylase alpha (*Acca*), whose products provide substrates for the biosynthesis of fatty acids, and an increase in the expression of carnitine palmitoyltransferase 1c (*Gpt1c*), essential for the transport of fatty acids into the mitochondria. These changes in gene expression therefore indicate that cellular energy stored in body fat might be low in these animals. It may also partly explain the reduced body weight of SHR compared with WKY observed in our previous study, due to reduced adipose tissue mass [7]. 

### Oxidative Stress Response

Four genes involved in stress resistance and detoxification have been found differentially expressed in young and old SHR. Retinol saturase (*Retsat*) as well as nitrilase family (*Nit*) and glutathione S-transferase (*Gst*) were underexpressed in SHR. This decrease may therefore adversely affect detoxification and allow oxidative damage to spread. Hsp40 implicated in various cellular functions, such as proper protein folding, protein transport, and response to stress was overexpressed in SHR. In contrast, in young SHR specifically, additional genes were found overexpressed: Glutathione peroxidase 1 (*Gp1*), known to protect organism form oxidative damage, and C-terminal binding protein (*Ctbp*), acting as a metabolic stress sensor [28], suggesting that there is an attempt to overcome the increased oxidative stress in young SHR. Beta-2 microglobuline and lectin overrepresentation, in addition to their roles in inflammation and intracellular traffic, respectively, is also associated with cellular stress in the brain.

### Response/Susceptibility to Ischemia 

In order to identify the contributors of the development of diffuse ischemic white matter lesions and lacunar infarcts characteristic of vascular dementia, a number of genes involved in the resistance and /or response to ischemia represent probably the best candidates. Glutathione S-transferase (Gst) detoxifies reactive oxygen species produced during brain ischemia. Decreased expression of Gst has been observed in SHR after stroke [29], as well as in the present study. It also suggests that brains of young and aged SHR suffer from chronic ischemia. Colony-stimulating factor-1 receptor (*Csf-1r*) that is weakly expressed by neurons in most area of the brain is upregulated in the area next to the ischemic lesion after ischemic injury [30]. CSF-1R is then expressed by neurons and microglia, and interacts with CSF produced by astrocytes, promoting neuronal survival. The CSF/CSF-1R signaling is therefore an important pathway between neurons, microglia and astrocytes. A defective expression of CSF or CSF-1R in the brain makes it considerably more vulnerable to ischemia [31]. The significant deficit in *Csf-1r* expression in 9-month-old SHR may therefore indicate a higher susceptibility of the tissue to hypoxia/ ischemia in these animals. Aggravation of neurological deficits by macrophage migration inhibitory factor (MIF) has been shown after experimental stroke and increases in MIF has been observed in cultured cortical neurons exposed to oxygen and glucose deprivation [32]. Therefore, the increased expression of *Mif* noted in 9-month-old SHR may point toward a low energy status in the brain tissue.

Furthermore, adenylate cyclase activating polypeptide 1 (*Acap1*) was overexpressed in 2-month-old animals. It is induced by chronic stress and may underlie the maladaptive remodeling and plasticity of brain regions associated with anxiety-like behavior [33].

### Hypertension Involved Genes

A number of genes differing in their expression level in SHR and supposed to confer hypertension have been characterized in kidneys [34,35]. However, except glutathione S-transferase (*Gst*), none of the revealed genes involved in the development of hypertension were found to be common in both analyses. Recently, evidence of the implication of only one renal gene (*Cd36*) predisposing to hypertension in SHR was clearly demonstrated [36]. Our comparison between the 2 and 9-month-old SHR may highlight additional genes involved in the development of hypertension, such as adducin-2b expressed more in 2-month-old than in 9-month-old SHR. Adducin-2b, *Add2*, was the unique gene showing changes in expression between 2- and 9-month-old SHR. A direct role of this protein in modulating arterial blood pressure was demonstrated using a adducin-2b-deficient mouse strain [37]. Indeed, the deficiency in adducin-2b induced hypertension, suggesting that in 9-month-old SHR, the decrease in gene expression may lead to an increase of blood pressure. 

### Apoptosis

As leukoaraiosis is an age-dependent degenerative condition caused by chronic ischemia, apoptosis in the white matter adjacent to leukoaraiosis lesions have already been demonstrated [38]. Several genes involved in the activation of apoptotic cell death have appeared differentially expressed in 9-month-old SHR only, but not in the younger 2-month-old animals. Two genes inducing cell death were found overexpressed in the oldest SHR: beclin1 (*Becn1*, and death effector domain-containing (*Dedd*). *Mif* can also be grouped in this class of genes, since the upregulation of MIF after experimental stroke was shown to promote cell death [32]. One gene exhibiting an anti-apoptotic action (ubiquitin-protein ligase E3) was found underexpressed in aged SHR compared to the younger SHR and to the age-matched WKY, clearly indicating a condition stimulating programmed cell death in the brain of hypertensive SHR. 

This study strongly suggests that young - yet pre-hypertensive - SHR suffer from chronic hypoxia-like conditions. The majority of genes that were expressed differently in SHR compared with age-matched WKY are known to respond to ischemia by altered expression. Several genes coding for mitochondrial complexes and functions, and for detoxifying and survival factors are also unsuitably expressed, rendering SHR more susceptible to ischemic injury and more vulnerable to high-energy consumption conditions. This molecular analysis therefore provides valuable new insights into the pathways accounting for the development of SVD. If these observations can be reproduced in human SVD, these findings might offer targets for preventive therapeutic options. Treatments aimed to improving brain energy metabolism and alleviating oxidative stress are definitely good candidates for this purpose.

## Figures and Tables

**Fig. (2):**
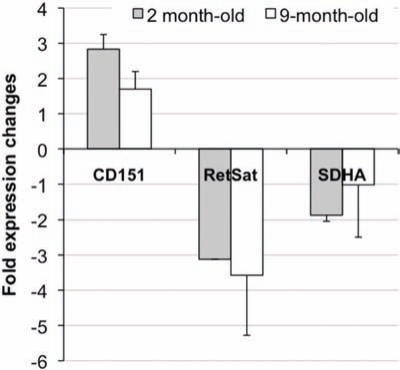
Real-time PCR analysis of selected mRNA found differentially expressed between SHR and WKY, common in the 2- and 9-month-old groups. Values are mean ± SD (n=4).

**Table 1.** Number of Significant Differentially Expressed Transcripts in the Frontal Cortex of 2- and 9-Month Old Spontaneously Hypertensive Rats (SHR) *versus* Age-Matched Normotensive Rats (WKY) (p<0.05, Fold Change <-2 or >2)

	2-month-old	9-month-old	Common in both
Number of genes Upregulated	58	69	10
Downregulated	98	62	7
Total	156	131	18^*^

* one gene was upregulated in 2-month-old SHR but downregulated in 9-month-old SHR versus age-matched WKY.

**Table 2.** List of the Differentially Expressed Genes Common in 2- and 9-Month-Old SHR *versus* Age-Matched WKY

Gene	Direction of change	*P* for 2-month-old	*P* for 9-month-old
**Endothelial proliferation, vascular development**			
CD151 antigen	Up in SHR	3.46E-04	2.58E-03
Activin A receptor	Up in SHR	7.31E-03	1.61E-03
p21 (CDKN1A)-activated kinase 2	Up in SHR	1.92E-03	5.03E-03
**Mitochondrial function**			
Cytochrome c oxidase	Up in SHR	4.39E-02	5.52E-02
Ribosomal protein L17	Down in SHR	1.73E-02	1.16E-03
**Energy and lipid metabolism**			
Succinate dehydrogenase complex a	Down in SHR	8.20E-04	3.44E-02
Acyl-coenzyme A dehydrogenase	Up in SHR	3.61E-03	9.35E03
**Oxidative stress**			
Retinol saturase (all-trans-13)	Down in SHR	3.45E-03	1.54E-03
Nitrilase family	Down in SHR	1.67E-03	6.77E-04
Hsp40 (DnaJ)	Up in SHR	8.51E-03	1.05E-03
Glutathione S-transferase	Down in SHR	1.74E-03	2.76E-03
**Protein catabolism,**			
Ubiquitin-activating enzyme E1	Up in SHR	1.94E-02	7.85E-04
Ubiquitin-protein ligase E3	Up in 2-month-old SHR Down in 9-month-old SHR	4.93E-04 -	- 4.67E-03
**Miscellaneous**			
Phosphatidylinositol glycan	Up in SHR	9.03E-05	4.20E-04
Translin-associated factor X	Up in SHR	2.55E-02	1.65E-03
Neuronal pentraxin receptor	Up in SHR	4.39E-02	3.62E-02
Secretogranin III	Down in SHR	3.87E-02	3.04E-03
Eukaryotic translation initiation factor 2C	Down in SHR	8.36E-03	3.14E-03

**Table 3.** List of the Differentially Expressed Genes in 2-Month-Old SHR *versus* Age-Matched WKY

Gene	Direction of change	*P* value
**Oxidative phosphorylation,**		
Cytochrome c oxidase subunit VII polypeptide 2	Up in SHR	4.39E-02
NADH dehydrogenase (ubiquinine) Fe-S protein 7	Up in SHR	1.66E-02
Mitochondrial ribosomal protein L18	Up in SHR	5.06E-03
Mitochondrial ribosomal protein L43	Down in SHR	3.15E-03
Mitochondrial ribosomal protein L48	Down in SHR	3.47E-03
Methylmalonyl coenzyme A mutase	Down in SHR	3.76E-04
Demethyl Q7	Up in SHR	1.28E-03
**Carbohydrate and lipid metabolisms**		
Brain glycogen phosphorylase	Up in SHR	4.91E-02
Acyl-coenzyme A dehydrogenase family member 9	Up in SHR	1.16E-03
Acyl-coenzyme A long-chain family member 3	Down in SHR	3.61E-03
Carnitine O-octanoyltransferase	Down in SHR	4.23E-03
**Amino acid metabolism**		
Branched chain ketoacid dehydrogenase kinase	Up in SHR	4.98E-04
Cystathionine beta synthase	Up in SHR	2.10E-03
Anti-oxidant and stress defenses		
Beta-2 microglobulin	Up in SHR	2.21E-03
Glutathione peroxidase 1	Up in SHR	5.29E-03
C-terminal binding protein	Up in SHR	5.05E-04
Lectin	Up in SHR	4.18E-04
**CNS development and function**		
Neuropilin and tolloid-like 1	Down in SHR	7.92E-03
Neuron navigator 3	Down in SHR	9.19E-03
Somatostatin	Down in SHR	9.66E-03
Syntaxin 7	Down in SHR	9.01E-03

**Table 4.** List of the Differentially Expressed Genes in 9-Month-Old SHR *versus* Age-Matched WKY

Gene	Direction of change	*P* value
**Oxidative phosphorylation**		
Cytochrome c oxidase subunit IV isoform 1	Up in SHR	5.52E-02
**Carbohydrate and lipid metabolisms**		
Carnitine palmitoyltransferase 1c	Up in SHR	3.49E-03
Acetyl-coenzyme A carboxylase alpha	Down in SHR	9.17E-03
Glycerol-3-phosphate acyltransferase	Down in SHR	7.03E-03
**Protein catabolism**		
Ubiquitin-activating enzyme E1-domain containing1	Up in SHR	7.85E-04
Praja2	Up in SHR	4.67E-03
Sumo2	Up in SHR	8.25E-03
Similar to Cezanne 2 protein	Down in SHR	2.53E-03
Dipeptidylpeptidase 10	Up in SHR	1.89E-04
**Apoptosis/cell death**		
Death effector domain-containing	Up in SHR	2.49E-03
Beclin 1	Up in SHR	7.17E-04
Macrophage migration inhibitory factor	Up in SHR	6.41E-03
Colony stimulating factor 1 receptor	Down in SHR	4.78E-04
**CNS structure and function**		
GPI deacetylase	Up in SHR	2.52E-04
Spectrin beta 3	Down in SHR	6.94E-04
Calpain 7	Up in SHR	1.45E-03
Neural stem cell-derived dendrite regulator	Down in SHR	8.33E-03
Kinesin family member 1A	Down in SHR	3.76E-04
Kv channel interacting protein 2	Up in SHR	7.26E-03
K invarding-rectifying channel	Up in SHR	8.65E-03
Glutamate receptor	Down in SHR	1.93E-03

**Table 5.** List of the Differentially Expressed Genes in 2- Month-Old SHR *versus* 9-Month-Old SHR

Gene	P value
***More in 2-month-old SHR***	
**Ion channels**	
Calcium channel alpha 1h	4.88E-03
Voltage-dependent anion channel 1	6.76E-03
Potassium voltage-gated channel	6.04E-03
**Nervous system development**	
Copine IV	7.28E-03
Neuronal growth regulator 1	3.27E-03
Development and differentiation enhancer	2.48E-03
**Hypertension-linked genes**	
Adducin 2 (beta)	8.42E-03
**Anti-apoptotic genes**	
PDZ domain containing RING finger 3	1.80E-03
***More in 9-month-old SHR***	
**Apoptosis-related genes**	
SCF apoptosis response protein 1	1.94E-03
Programmed cell death protein 11	1.63E-03
Septin 4	2.34E-03
